# Machine Learning-Assisted Measurement of Three-Dimensional Particle Self-Rotation and Revolution in Swirling Flow Fields

**DOI:** 10.3390/s26196063

**Published:** 2026-09-24

**Authors:** Fengqin He, Weiqing Liu, Yuan Huang, Qibin Liu

**Affiliations:** 1 The College of Information, Mechanical and Electrical Engineering, Shanghai Normal University, Shanghai 200234, China; liuweiqing147@gmail.com (W.L.); qibin.liu9812@outlook.com (Q.L.); 2 Institute of Environmental Pollution and Health, School of Environmental and Chemical Engineering, Shanghai University, Shanghai 200444, China; 3 Shangtex Investment & Trading Co., Ltd., Shanghai 201306, China

**Keywords:** three-dimensional motion sensing, machine vision, deep learning, object tracking, hydrocyclone, particle dynamics

## Abstract

Accurate characterization of particle dynamics in complex swirling flows is essential for understanding flow behavior and transport mechanisms. However, extracting three-dimensional (3D) rotational motion parameters of particles from High-Speed Motion Analyzer (HSMA) imaging experiments remains challenging due to the limitations of conventional manual frame-by-frame analysis, which suffers from low efficiency, subjective errors, and poor scalability. In this study, an intelligent imaging-based sensing system is proposed for automated 3D measurement of coupled particle self-rotation and revolution motion in hydrocyclone swirling fields. The proposed system integrates dual orthogonal high-speed cameras with a machine learning-driven processing framework, enabling an end-to-end workflow consisting of particle detection, multi-object tracking, motion-state recognition, kinematic parameter calculation. To achieve robust particle detection under different imaging conditions, You Only Look Once version 8 (YOLOv8) detectors are trained following a mixed-view strategy: the Y-view detector is trained on the Y-camera data, whereas the X-view detector is trained on the merged dual-camera data. ByteTrack is subsequently employed to associate particle detections across consecutive frames and generate continuous trajectories. For motion-state recognition, EfficientNet-B1 is adopted to classify the relative positional states of internal particle markers. Furthermore, a Self-Attention Generative Adversarial Network (SAGAN) is introduced for minority-class sample generation to alleviate class imbalance and improve classification robustness.Based on the extracted trajectories and state information, a kinematic model is integrated to automatically estimate particle revolution and self-rotation velocities. Experimental results demonstrate that the proposed YOLOv8 detectors achieve reliable detection performance, with mean average precision at an Intersection over Union (IoU) threshold of 0.5 (mAP@0.5) values of 79.84% and 71.62% for the two imaging views, respectively. The combined YOLOv8-ByteTrack framework achieves Higher Order Tracking Accuracy (HOTA) and Multiple Object Tracking Accuracy (MOTA) values of 63.791 and 90.49, respectively. After SAGAN-based data augmentation, the classification accuracy of EfficientNet-B1 increases from 98.62% to 99.54%. System-level validation using more than 60 randomly selected particle trajectories shows average measurement accuracies of 97.59% for revolution velocity and 91.8% for self-rotation velocity, while reducing the processing time from more than 600 s manually to 11.1 s per trajectory. The proposed machine learning-assisted particle 3D motion sensing system provides an efficient and reliable solution for automated extraction of particle kinematic information under complex flow imaging conditions, offering new opportunities for intelligent monitoring and mechanism analysis of multiphase flow systems.

## 1. Introduction

Particle dynamics in complex flow fields provide essential information for understanding multiphase transport phenomena, fluid-particle interactions, and process performance. Accurate measurement of particle motion parameters, including revolution velocity, self-rotation velocity, and three-dimensional spatial coordinates, is critical for revealing particle migration mechanisms and developing intelligent sensing and monitoring strategies for industrial flow systems [1,2,3]. However, due to the highly transient nature, nonlinear characteristics, and complex three-dimensional structures of multiphase flows, reliable acquisition of particle motion information remains challenging under conditions involving strong turbulence, particle interactions, and high-speed motion.

Hydrocyclones represent typical complex swirling flow systems in which particle motion is governed by the coupled effects of centrifugal force, drag force, pressure gradients, and turbulent fluctuations. Owing to their simple structure, absence of moving components, high processing capacity, and low energy consumption, hydrocyclones have been widely applied in mineral processing, petrochemical engineering, environmental remediation, and other industrial fields for solid-liquid separation, classification, and purification processes [4,5]. As a representative multiphase swirling system, the internal flow of hydrocyclones involves strong rotational motion, axial backflow, and radial migration within a highly unsteady three-dimensional flow field. Under such complex conditions, particles experience various dynamic behaviors, including self-rotation, revolution, aggregation, and separation, which determine their trajectories and transport characteristics. Therefore, accurate sensing and quantitative characterization of particle dynamics in hydrocyclone swirling fields are essential for understanding flow-particle interaction mechanisms, and improving the controllability of industrial multiphase flow processes.

High-speed micro-imaging [6,7,8] provides an effective method to capture transient particle motion with high temporal resolution. Nevertheless, converting high-speed image sequences into quantitative three-dimensional particle kinematics remains difficult. The difficulty is not simply associated with particle detection, but arises from several interconnected sensing problems. First, particle trajectories are observed from two-dimensional image planes, whereas the actual particle motion occurs in a three-dimensional swirling field. Therefore, complementary imaging views and appropriate geometric relationships are required to recover spatial motion information. Second, rapid particle displacement, overlap, and motion blur may lead to missed detection and trajectory discontinuities, making reliable trajectory extraction difficult under high-speed operating conditions. Third, particle self-rotation is not directly observable as a continuous rotational variable from a single image. Instead, the self-rotation information must be inferred from the temporal variation in internal tracer-marker configurations. These characteristics indicate that reliable particle motion sensing requires a coordinated processing chain that connects multi-view image acquisition, particle localization, trajectory association, motion-state recognition, and physical kinematic reconstruction.

Recent advances [9] in machine learning and computer vision have provided new opportunities for intelligent image-based sensing and automated analysis of complex flow phenomena. Advanced vision algorithms, including object detection [10,11], multi-object tracking [12], and image-based state recognition, have demonstrated significant potential for extracting quantitative information from dynamic visual data. However, most existing studies have primarily focused on object localization and two-dimensional trajectory tracking, flow visualization, or qualitative motion analysis. In comparison, an integrated sensing methodology that converts multi-view high-speed images into quantitative measurements of coupled particle revolution and self-rotation in a three-dimensional swirling flow remains insufficiently developed. In particular, the connection between visual observations and physically interpretable kinematic parameters requires not only robust image analysis but also an appropriate physical reconstruction model.

To address this limitation, this study develops a machine learning-assisted three-dimensional particle motion sensing system based on dual-orthogonal high-speed micro-imaging. The objective is not to introduce new individual machine learning architectures, but to establish a task-oriented sensing methodology that integrates complementary visual observations, machine learning-based perception, and physical kinematic reconstruction. The proposed framework comprises sequential modules for particle detection, multi-object tracking, motion-state recognition, and kinematic parameter estimation, thereby establishing a continuous information pathway from high-speed particle images to quantitative motion parameters, including 3D particle coordinates, revolution velocity, and self-rotation velocity. Information from orthogonal imaging views is further combined with geometric and kinematic constraints to reconstruct the three-dimensional motion state of individual particles. By coupling data-driven visual perception with physics-based kinematic modeling, the framework converts complex high-speed image observations into reproducible and physically interpretable particle motion information.

The proposed approach extends the capability of high-speed micro-imaging from conventional qualitative visualization to automated and quantitative particle motion sensing. By jointly characterizing particle position, revolution velocity, and self-rotation velocity, the framework provides a more comprehensive description of particle dynamics in complex swirling flow fields. This capability provides a practical basis for investigating particle-flow interactions, particle migration, and separation mechanisms, while offering a potential sensing and monitoring tool for data-driven analysis of industrial multiphase flow processes.

## 2. Experiment and Methods

### 2.1. Machine Learning-Assisted Three-Dimensional Sensing System

The experimental and computational procedures used to implement the proposed sensing framework are summarized in Figure 1. The main equipment information used in the experiments is summarized in Table 1. Any equipment not listed in the table was custom-designed and developed in-house by the authors’ research group. The proposed framework establishes an end-to-end sensing pipeline integrating HSMA-based data acquisition, machine learning-driven visual perception, and kinematic parameter estimation. The pipeline comprises four sequential modules: HSMA experiment, particle detection and multi-object tracking, motion-state recognition, and kinematic parameter calculation.

### 2.2. HSMA Experimental Configuration

To investigate particle dynamics under different swirling flow conditions, HSMA-based experiments were performed in a hydrocyclone. Highly uniform tracer microspheres were fabricated using a two-stage glass capillary microfluidic device according to a previously reported method [13]. The microspheres consisted of a transparent spherical shell with two embedded black marker cores, enabling visual characterization of particle rotational motion. The average diameter of the microspheres was approximately 470 μm. The internal marker structure provided identifiable features for tracking both particle orientation variation and spatial displacement during motion. The experimental setup consists of a hydrocyclone experimental system shown in Figure 2a and a high-speed imaging system shown in Figure 2b. The quartz-glass hydrocyclone with a cylindrical section diameter of 25 mm served as the core component of the experimental platform for generating the swirling flow field and inducing particle motion. Tracer microspheres were introduced into the hydrocyclone through a tangential inlet together with water, generating a highly rotational three-dimensional swirling flow field. To capture the particle dynamics from complementary perspectives, two high-speed cameras were arranged along mutually orthogonal viewing directions to simultaneously record the particle motion inside the hydrocyclone. Both cameras were equipped with macro lenses (AF Micro-NIKKOR 60 mm f/2.8D). The X-view camera operated at 4000 fps with an image resolution of 640 × 360 pixels, while the Y-view camera operated at 8000 fps with a resolution of 1024 × 768 pixels. Two light sources were positioned to provide adequate and uniform illumination for high-speed imaging. For spatial calibration, a standard graduated ruler was placed inside the field of view of each camera as a spatial reference. By extracting the pixel length of the ruler divisions from the recorded images, the pixel-to-physical scale factor of each view was determined directly, so that no complex intrinsic or extrinsic camera calibration was required; the image coordinate system could thus be converted into physical dimensions. Detailed information on the high-speed imaging experiments is provided in the referenced study [13]. The synchronized orthogonal imaging system provided multi-view information for subsequent particle detection, trajectory tracking, motion-state recognition, and kinematic parameter estimation.

### 2.3. Methodology

A HSMA experiment generates a large number of image sequences containing microsphere motion information. To overcome the limitations associated with manual data process, this study proposes an advanced deep learning-based particle motion sensing workflow, as illustrated in Figure 3.

#### 2.3.1. Object Detection and Tracking

YOLO family [14,15] has become one of the representative approaches for real-time object localization because of its favorable balance between detection accuracy and computational efficiency. Recent developments in YOLO-based detection have further improved the capability to identify small and densely distributed targets through multi-scale feature fusion, attention mechanisms, improved detection heads, and more efficient network architectures [10,11]. Nevertheless, small-object detection remains challenging because particles may occupy only a limited number of pixels and contain insufficient discriminative spatial features. In high-speed micro-imaging, this problem is further aggravated by motion blur, illumination variation, complex background textures, and particle overlap. Although newer YOLO variants, such as YOLOv10, have continued to improve the accuracy-efficiency trade-off through architectural and post-processing optimization, their advantages are mainly established on general-purpose benchmark datasets [16]. For the present task, therefore, detector selection should consider not only the nominal benchmark performance but also robustness, implementation maturity, computational efficiency, and adaptability to the specific imaging conditions of microscale particles. In this study, YOLOv5s, YOLOv8s, YOLOv9s, and RT-DETR-L (Ultralytics 8.4.149) were considered as representative candidate detectors and comparatively evaluated under the particle imaging conditions investigated here.

Reliable multi-object tracking is essential for associating particle observations across consecutive frames and reconstructing continuous particle trajectories. Conventional tracking-by-detection methods, such as Simple Online and Realtime Tracking (SORT) [17], primarily rely on motion prediction and data association, whereas subsequent approaches have introduced improved motion models and additional information to enhance robustness under occlusion and nonlinear motion. ByteTrack [18] proposed an effective association strategy that considers both high- and low-confidence detections, enabling the recovery of objects that might otherwise be discarded because of temporary occlusion or degraded detection confidence [12]. More recent approaches, such as Observation-Centric SORT (OC-SORT) [19], further improve tracking robustness by exploiting observation-based information to correct motion-estimation errors, particularly in the presence of occlusion and nonlinear motion [20]. These developments demonstrate that robust trajectory association is a critical component of particle motion sensing, especially when particles move rapidly, overlap with neighboring particles, or are affected by motion blur. However, most existing tracking methods have been developed and evaluated primarily on natural-scene benchmarks, where targets generally contain richer spatial and appearance information than microscale particles. Their effectiveness under high-speed particle imaging conditions therefore requires task-specific evaluation. In the present study, multiple object detection and tracking configurations were comparatively evaluated to identify a suitable visual perception strategy for small-particle imaging. In particular, YOLO-based particle detection was combined with representative tracking algorithms, including ByteTrack and OC-SORT, and their performance was experimentally compared in terms of trajectory association and tracking reliability under the imaging conditions considered in this work.

All models were trained and evaluated using their default hyperparameter settings, as provided in their respective frameworks, unless otherwise specified. The model training and evaluation experiments described in this subsection were conducted on an NVIDIA Tesla P100-PCIE GPU (16 GB) with Python 3.12.12, PyTorch 2.8.0+cu126, and CUDA 12.6.

##### Data Preprocessing

The video frames were preprocessed using grayscale conversion and contrast enhancement to ensure the trajectories of particles in high-speed turbulence were clearly visible. The position and state of each particle in every frame were annotated, and a fundamental training dataset was constructed to support the subsequent study of particle motion behavior. Specifically, A total of 508 images containing multiple annotated particles moving within the hydrocyclone were used as the training set. The annotated sequences were divided into training, validation, and test subsets at a ratio of 8:1:1 (80% for training, 10% for validation, and 10% for testing) as follows: the frames of each camera sequence were sorted in acquisition order and partitioned into contiguous time segments, i.e., the first 80% for training, the next 10% for validation, and the last 10% for testing, and a 10-frame buffer was discarded at each segment boundary so that no temporally adjacent frames were assigned to different subsets. For the Y-camera sequence, the resulting training, validation, and test sets contain 508, 55, and 65 images, respectively, and for the X-camera sequence, they contain 278, 26, and 36 images, respectively. The full experimental database comprises more than 80 GB of high-speed image sequences acquired at five inlet flow rates (450, 550, 650, 750, and 850 L/h) and several hydrocyclone axial heights, with approximately 10,000 frames recorded for each inlet flow rate. The annotated images used for detector training were acquired at an inlet flow rate of 550 L/h, under which particle images are the clearest; the system-level evaluation stage additionally covered the other inlet flow rates and multiple axial heights. Each image contained approximately four to eight annotated particles, with manual annotation applied to ensure accuracy and consistency.

##### Training and Evaluation

As demonstrated in Table 2, RT-DETR-L achieved the highest detection accuracy among the evaluated frameworks, with a precision of 87.15%, a recall of 90.15%, and an mAP@0.5 of 87.87%, but required the longest inference time (35.04 ms). YOLOv8s provided the most favorable balance between detection accuracy (mAP@0.5 of 79.84%) and inference efficiency (14.36 ms) among the compared frameworks, and was therefore adopted in the proposed sensing framework. These results were obtained on a common test set consisting of 65 images, and they demonstrate the effectiveness of the adopted detector in localizing small particles. The competitive detection accuracy of YOLOv8s can be attributed to its architectural advancements, including the Cross Stage Partial (C2f) module, anchor-free design, decoupled head, and optimized loss functions such as Complete Intersection over Union (CIoU) [21] and Distribution Focal Loss (DFL) [22,23,24]. These enhancements make YOLOv8 particularly well-suited for detecting small particles with an average size of 8 × 8 pixels. During training, YOLOv8 was initialized with pretrained weights from yolov8s.pt and fine-tuned over 100 epochs with a batch size of 16 images and an input resolution of 640 × 640 pixels. All detectors compared in this section were trained and evaluated under the same chronologically split protocol described in the Data Preprocessing paragraph, with identical code and hyperparameters (ultralytics 8.4.149, seed = 42, 100 epochs, input size 640 × 640, batch size 16, and evaluation threshold conf = 0.25). During detection, non-maximum suppression was applied with the default IoU threshold of 0.7.

Furthermore, Table 3 highlights the tracking performance of different model-algorithm combinations. The YOLOv8-ByteTrack combination achieved a HOTA value of 63.791 and a MOTA value of 90.49 [25,26], significantly outperforming the YOLOv8-OC-SORT combination, which attained scores of 50.2 and 64.986, respectively. This improvement can be attributed to ByteTrack’s strategy of associating both high-confidence and low-confidence detections, where high-confidence detections provide reliable object identities while low-confidence detections help recover temporarily lost targets, thereby improving multi-object tracking robustness [12]. In particular, the low-confidence detections are especially important when tracking small objects, as their size often results in lower detection confidence, making robust association mechanisms like ByteTrack crucial for maintaining tracking precision. The evaluations were conducted using a dataset consisting of 276 consecutive images. These 276 consecutive frames serve a distinct validation role: they are reserved exclusively for evaluating the trajectory-association capability of the tracking module and are not used for detector training; the detector training sets (508 Y-camera images and 278 X-camera images, as described in the Data Preprocessing paragraph) and the tracking evaluation sequence therefore constitute independent validation tasks.

#### 2.3.2. Particle State Classification

Following particle detection and trajectory association, particle-state classification is a key step toward the quantitative characterization of particle motion. Object detection determines particle locations, trajectory association establishes temporal correspondence across consecutive frames, while state classification further identifies subtle morphological and positional changes in image sequences, providing the basis for characterizing particle self-rotation. Considering the small particle size and high frame rate of the image sequences, the classification backbone should provide sufficient feature representation capability while maintaining computational efficiency. To meet these requirements, various general-purpose image classification backbones have been compared for visual recognition tasks. Residual Network (ResNet) introduced residual connections to facilitate the optimization of deep networks and has become a widely used baseline for visual recognition [27]. Gated Convolutional Networks with Hybrid Connectivity (HCGNet) further combines global dense connectivity, local residual connections, and gated feature fusion to reduce feature redundancy while maintaining strong representation capability [28]. EfficientNet adopts compound scaling to jointly balance network depth, width, and input resolution, providing a favorable trade-off between classification accuracy and computational cost [29]. These architectures represent different strategies for balancing representation capacity and computational efficiency. For the present particle-state recognition task, where the target images are small and the processing pipeline involves high-speed image sequences, a lightweight backbone with sufficient feature extraction capability is desirable.

##### Data Preprocessing

Particle-only regions were extracted from video frames, resulting in a dataset specifically designed for target recognition and classification tasks. This dataset initially consisted of 1234 images of the separated state and 353 images of the aggregated state. To address the imbalance in the dataset, a Self-Attention Generative Adversarial Network (SAGAN) was employed to generate 576 additional images of the aggregated state [30,31]. This augmentation effectively balanced the dataset and improved the classification accuracy to 99.54% (Table 4).

##### Training and Evaluation

As shown in Table 5, the combination of YOLOv8 detection with EfficientNet-B1 classification achieved a combined precision of 84.23% and a combined recall of 83.20%, which is the most balanced performance among the compared methods: it substantially outperformed the combination of YOLOv8 detection with the OpenCV-based rule method (73.77%/72.16%), and provided a precision comparable to Mask R-CNN (84.90%) with a much higher recall (63.70%). EfficientNet-B1 was selected as the classification model due to its superior accuracy and computational efficiency [32]. Specifically, the EfficientNet-B1 variant was implemented with PyTorch and initialized with ImageNet pretrained weights; the input images were resized to 224 × 224 pixels and normalized with μ=[0.485,0.456,0.406] and σ=[0.229,0.224,0.225]. The classifier was fine-tuned for 150 epochs using the Adam optimizer with an initial learning rate of $1\times 10^{-3}$ and a batch size of 32, and the checkpoint with the best validation accuracy was retained. On a test set comprising 222 aggregated state images and 214 separated state images, the model achieved a classification accuracy of 99.54%. In comparison, the OpenCV-based state classification method utilized contour detection to extract minimum enclosing rectangles from particle contours in binarized images. By analyzing the aspect ratio of these rectangles, particles were classified as dispersed if their aspect ratio exceeded a predefined threshold, otherwise, they were classified as aggregated. While computationally lightweight, this method struggled with irregular particle shapes and overlapping structures, leading to lower accuracy.

#### 2.3.3. Calculation of Particle Velocities

To elucidate the underlying motion mechanisms of microspheres in the three-dimensional swirling flow field, it is essential to obtain accurate information on their trajectories and dynamic behaviors during flow transport. Among these dynamic characteristics, the self-rotation and revolution motions of microspheres provide important indicators for understanding particle-flow interactions. When the self-rotation axis of a microsphere is aligned with the camera imaging plane, the internal core markers exhibit periodic transitions between overlapping and separated configurations in consecutive image frames. This periodic variation enables the determination of microsphere self-rotation speed through image processing, with a minimum detectable angular displacement of π/2. The self-rotation speed is calculated by counting the number of marker-state transitions according to Equation (1).

Due to the stochastic nature of microsphere motion within the swirling flow field, only a limited number of microspheres remain close to the hydrocyclone wall during revolution. For these particles, the revolution radius can be directly determined from the wall boundary. However, most microspheres migrate along trajectories at different radial positions from the wall, making direct determination of the revolution radius challenging. Therefore, accurate measurement of the radial distance between the microspheres and the hydrocyclone wall is required to obtain reliable revolution radius estimations. In this study, the distance between particle and wall was recorded by Camera X. The calculation methods for microsphere revolution velocity in the hydrocyclone are described in Equations (2)–(5), and the corresponding parameters are illustrated in Figure 4 and Table 6.

(1) $$\omega_{z}=\frac{n\pi f_{y}}{2(N_{f}-1)}$$

(2) $$h_{\mathrm{avg}}=\frac{h_{\mathrm{start}}+h_{\mathrm{end}}}{2}$$

(3) $$D_{z}=D\left(h_{\mathrm{avg}}\right)$$

(4) $$r=\frac{D_{z}}{2}-\delta_{r}$$

(5) $$\omega_{\theta }=\frac{f_{y}}{N_{f}-1}\left(\sin^{-1}\frac{L_{1}}{r}+\sin^{-1}\frac{L_{2}}{r}\right)$$

## 3. Results and Discussion

### 3.1. Reliability of the Integrated Motion Sensing Framework

The reliability of the proposed sensing framework was systematically assessed from three perspectives: particle localization accuracy, trajectory reconstruction performance, and motion-state recognition capability. These three modules collectively establish the information pathway from high-speed micro-images to quantitative particle kinematic parameters.

The YOLOv8-based detection models demonstrated reliable particle localization under the two orthogonal imaging conditions. As summarized in Table 7, the Y-view model achieved a recall of 83.58% and an mAP@0.5 value of 79.84% on the Y-camera test set, while the X-view model achieved a recall of 82.49% and an mAP@0.5 value of 71.62% on the merged dual-camera test set. The lower precision of the X-view model was mainly associated with the lower image resolution and the smaller apparent particle size in the X-camera view, as well as the background complexity. Nevertheless, the mixed-view training strategy provided stable particle localization for subsequent trajectory analysis.

Based on the detected particle positions, the YOLOv8-ByteTrack framework was employed to reconstruct continuous particle trajectories. As shown in Table 2, compared with the YOLOv8-OC-SORT framework, the proposed method achieved superior tracking performance, with HOTA and MOTA values of 63.791 and 90.49, respectively. These data demonstrate that the proposed tracking strategy can effectively maintain trajectory continuity and identity consistency under high-speed micro-imaging conditions. As shown in Figure 5, the visualization results further validate the reliability of the proposed tracking strategy for extracting stable particle trajectories from high-speed micro-imaging sequences.

The extracted trajectories were further processed by the EfficientNet-B1-based state recognition module to identify the marker configurations associated with particle self-rotation. The incorporation of SAGAN-generated minority-class samples improved the classification accuracy from 98.62% to 99.54%, indicating enhanced recognition capability for less-represented particle states. The accurate identification of marker-state transitions provides reliable intermediate information for subsequent calculation of particle rotational dynamics.

Overall, the integrated YOLOv8-ByteTrack-EfficientNet B1 pipeline successfully converts raw high-speed imaging sequences into reliable trajectory and state information, establishing a robust foundation for automated estimation of particle self-rotation and revolution velocities.

### 3.2. System-Level Validation of the Proposed Sensing Framework

To further evaluate the reliability of the proposed machine learning-assisted sensing framework, more than 60 particle trajectories were randomly selected for system-level validation. These trajectories were not drawn from a single operating condition: they cover the five inlet flow rates (450–850 L/h) and multiple hydrocyclone axial heights used in the experiments. The particle motion parameters obtained from the automated framework were compared with manually processed results, including both revolution velocity and self-rotation velocity measurements. Figure 6 presents the comparison between the automated measurements and manual results under different inlet flow rates and hydrocyclone operating heights. In the caption, THIS STUDY denotes the particle motion measurement results obtained using the proposed machine learning-based framework, whereas MANUAL denotes the corresponding reference results obtained through manual data processing analysis of the experimental images. Both results were derived from the comprehensive experimental database collected in this study. These manually obtained results were used as the reference measurements for evaluating the accuracy and generalization of the proposed framework under different inlet flow rates and axial positions. The proposed framework achieved an average accuracy of 97.59% for revolution velocity, demonstrating excellent agreement with manual measurements. For self-rotation velocity estimation, the average accuracy reached 91.8%. The slightly lower accuracy compared with revolution velocity was mainly attributed to the inherent dependence of self-rotation measurement on the visibility of internal marker-state transitions. Under higher flow rates and larger hydrocyclone heights, intensified turbulence and increased particle motion blur reduced the clarity of marker configurations, resulting in larger uncertainties in self-rotation velocity estimation. As summarized in Figure 6f, the per-condition accuracy of self-rotation velocity estimation ranges from 88.8% to 95.0% across the five inlet flow rates, while the revolution velocity accuracy remains stable between 96.4% and 97.9%, demonstrating consistent measurement performance across operating conditions. Compared with conventional manual frame-by-frame analysis, the proposed machine learning-assisted framework significantly reduces processing time and eliminates operator-dependent subjective variations while maintaining high measurement accuracy. These results demonstrate the potential of the developed system for efficient, reproducible, and large-scale quantitative characterization of particle dynamics in complex swirling flow fields.

### 3.3. Quantitative Characterization of Particle Motion Dynamics Under Swirling Flow Conditions

The proposed automated sensing framework enables quantitative characterization of particle motion behavior under different inlet flow conditions. Figure 7 presents the distribution characteristics of particle self-rotation and revolution velocities under different inlet flow rates and hydrocyclone axial heights. The results demonstrate that both self-rotation and revolution velocities exhibit an approximately linear increasing trend with increasing inlet flow rate. This behavior is consistently observed at different measurement heights inside the hydrocyclone, indicating that higher inlet flow rates enhance momentum transfer from the swirling flow to the particles, resulting in intensified self-rotation and revolution motion. The results further reveal that particle self-rotation and revolution velocities exhibit similar responses to inlet flow rate but different spatial variations along the hydrocyclone height. For revolution velocity, different variation patterns are observed in the cylindrical and conical sections. In the cylindrical section (h<3.5 cm), the revolution velocity gradually decreases with increasing height, which may be related to viscous dissipation and the redistribution of angular momentum during swirling flow development. In contrast, in the cone section (h>3.5 cm), the revolution velocity increases with height due to the decreasing flow radius and enhanced conservation of angular momentum. A similar non-monotonic variation is observed for particle self-rotation velocity, which initially decreases and subsequently increases along the axial direction. This trend is particularly evident at an inlet flow rate of 850 L/h, where the transition between the two regions is more pronounced. The increase in revolution velocity within the small-radius conical region may enhance particle rotational motion through stronger centrifugal coupling and flow-particle interaction. These results demonstrate that the developed sensing framework can effectively capture spatial variations in particle dynamics and provide quantitative insights into the coupling between swirling flow structures and particle motion behavior.

Particle self-rotation is governed not only by local velocity gradients but also by particle position and instantaneous particle-fluid and particle-air interactions. Consequently, variations in the motion of individual particles may still occur under the same inlet flow rate. The relatively flat velocity distribution observed at an inlet flow rate of 750 L/h may be related to the flow-field characteristics under this operating condition. Under these experimental conditions, no obvious air core was visually observed, and air bubbles were rarely present within the hydrocyclone, which was predominantly filled with liquid and exhibited a relatively stable, liquid-dominated swirling flow structure. Although individual particle motions may still fluctuate owing to differences in their instantaneous positions and local interactions, the relatively stable bulk flow structure under this condition may result in a comparatively flat overall velocity distribution.

### 3.4. Reliability and Measurement Capability of the Proposed Sensing Framework

The proposed machine learning-assisted sensing framework establishes an automated pathway from high-speed micro-imaging data to quantitative particle motion parameters. By integrating particle detection, trajectory reconstruction, motion-state recognition, and physical kinematic modeling, the developed system overcomes the limitations of conventional manual frame-by-frame analysis and enables reproducible measurement of three-dimensional particle dynamics under complex swirling flow conditions.

The reliability of the proposed sensing strategy was demonstrated through system-level validation against manual data processing. Most of the relevant studies [33,34,35] on particle self-rotation in flow fields have relied on manual data-processing methods; therefore, manual measurements were adopted as the reference for evaluating the proposed automated framework. During manual processing, the largest measurement uncertainty was associated with the determination of the particle self-rotation angle. The uncertainty in manually determining the self-rotation angle was estimated to be no greater than 45°. Since the observed microspheres generally underwent a self-rotation of at least 180°, the corresponding maximum relative uncertainty was estimated to be approximately 25%. Against this manual reference, the automated framework achieved average accuracies of 97.59% and 91.8% for revolution velocity and self-rotation velocity estimation, respectively. These results indicate that the proposed machine learning-based image interpretation framework can provide reliable quantitative measurements of particle dynamics while substantially reducing the need for manual data processing.

In addition to measurement accuracy, the proposed framework provides significant advantages in processing efficiency and reproducibility. The comparison of processing time between manual analysis and the automated approach is summarized in Table 8. The average processing time per particle trajectory was reduced from more than 600 s using conventional manual analysis to only 11.1 s with the proposed framework, demonstrating a substantial improvement in high-speed image data processing efficiency. Furthermore, the automated workflow minimizes operator-dependent subjective variations and ensures consistent analysis results across different experimental conditions. The capability of batch processing large-scale trajectory data further enhances the scalability of the proposed system, enabling systematic investigation of particle motion behaviors under various operating conditions. Overall, the developed sensing framework extends high-speed micro-imaging from conventional qualitative observation toward automated and quantitative particle motion sensing. By providing efficient, reproducible, and scalable measurement of particle dynamics, this approach offers a valuable tool for investigating particle-flow interactions and supporting mechanism analysis and optimization of complex swirling flow systems.

### 3.5. Limitations and Future Works

Despite the promising experimental results, the proposed framework was developed and validated primarily on a hydrocyclone experimental platform. Further validation across different flow systems and operating conditions would help establish its broader applicability. Future work will therefore focus on improving the robustness of the visual perception modules and evaluating model adaptation and generalization under a wider range of experimental conditions.

## 4. Conclusions

In this study, a machine learning-assisted three-dimensional particle motion sensing framework was developed for automated characterization of particle self-rotation and revolution dynamics in hydrocyclone swirling fields. By integrating dual orthogonal high-speed micro-imaging with machine learning-based visual perception and kinematic modeling, the proposed framework establishes an end-to-end workflow from raw image acquisition to quantitative particle motion parameter estimation. The proposed framework achieved high consistency with manual measurements, with average estimation accuracies exceeding 90% for both revolution and self-rotation velocities. The main contributions of this study are summarized as follows:

1. An end-to-end three-dimensional particle motion sensing framework was established by integrating dual orthogonal high-speed micro-imaging, machine learning-based analysis, and kinematic modeling, enabling automated transformation from particle images to quantitative motion parameters;
2. A robust visual perception strategy based on YOLOv8, ByteTrack, and EfficientNet-B1 was developed for complex swirling flow environments, enabling accurate particle detection, trajectory association, and motion-state recognition under challenging conditions involving small targets, motion blur, trajectory discontinuity, and class imbalance.
3. A quantitative kinematic reconstruction approach was developed to characterize three-dimensional particle motion behaviors, enabling systematic analysis of the coupling between swirling flow structures and particle dynamics under different operating conditions.

Overall, the proposed machine learning-assisted sensing framework provides an efficient and reliable approach for quantitative characterization of particle dynamics in hydrocyclone swirling flows. The developed methodology extends high-speed micro-imaging from conventional qualitative observation toward automated and reproducible particle motion sensing, providing valuable support for particle-flow interaction analysis, swirling flow mechanism investigation, and optimization of industrial separation processes.

## Figures and Tables

**Figure 1:**
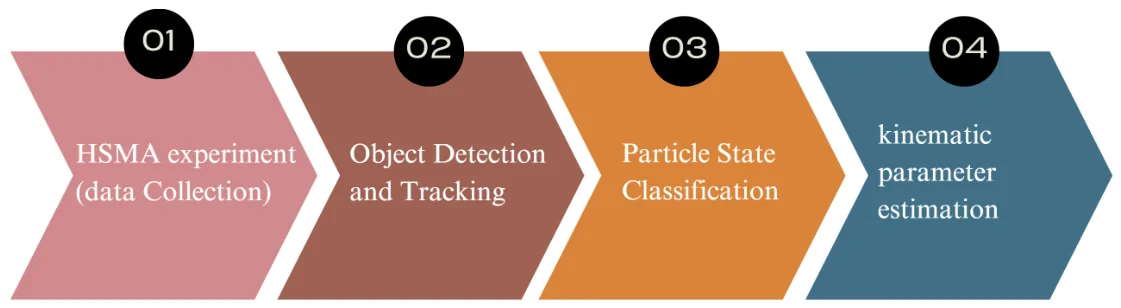
Sensing system framework. The four colored, arrow-shaped blocks correspond to the four sequential modules of the pipeline (numbered 01–04): HSMA experiment (data collection), object detection and tracking, particle state classification, and kinematic parameter estimation; the block shapes indicate the processing order.

**Figure 2:**
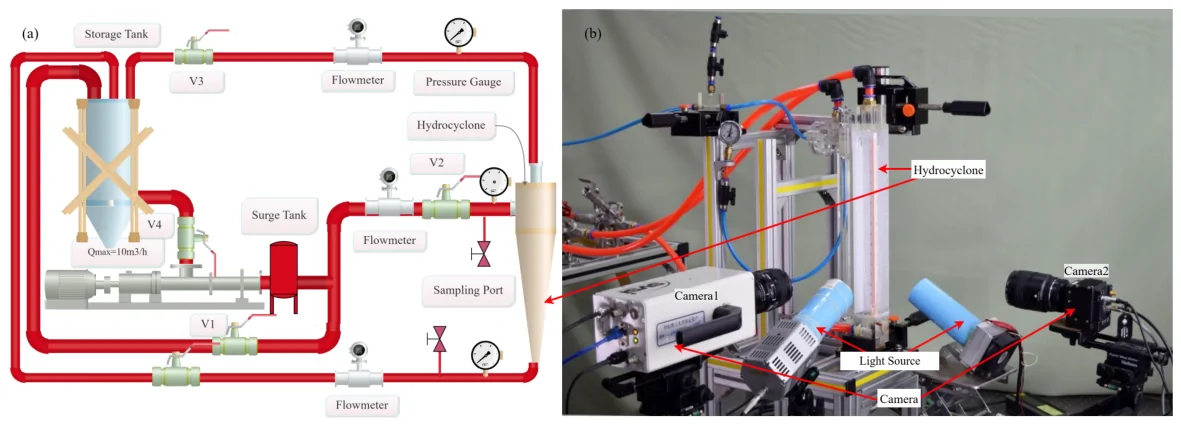
Experiment platform. (**a**) Schematic diagram of hydrocyclone experimental system, showing the storage tank, pump, surge tank, flowmeters, pressure gauges, control valves, and the hydrocyclone; (**b**) HSMA experimental setup, including the transparent hydrocyclone, the two orthogonally arranged high-speed cameras, and the illumination system.

**Figure 3:**
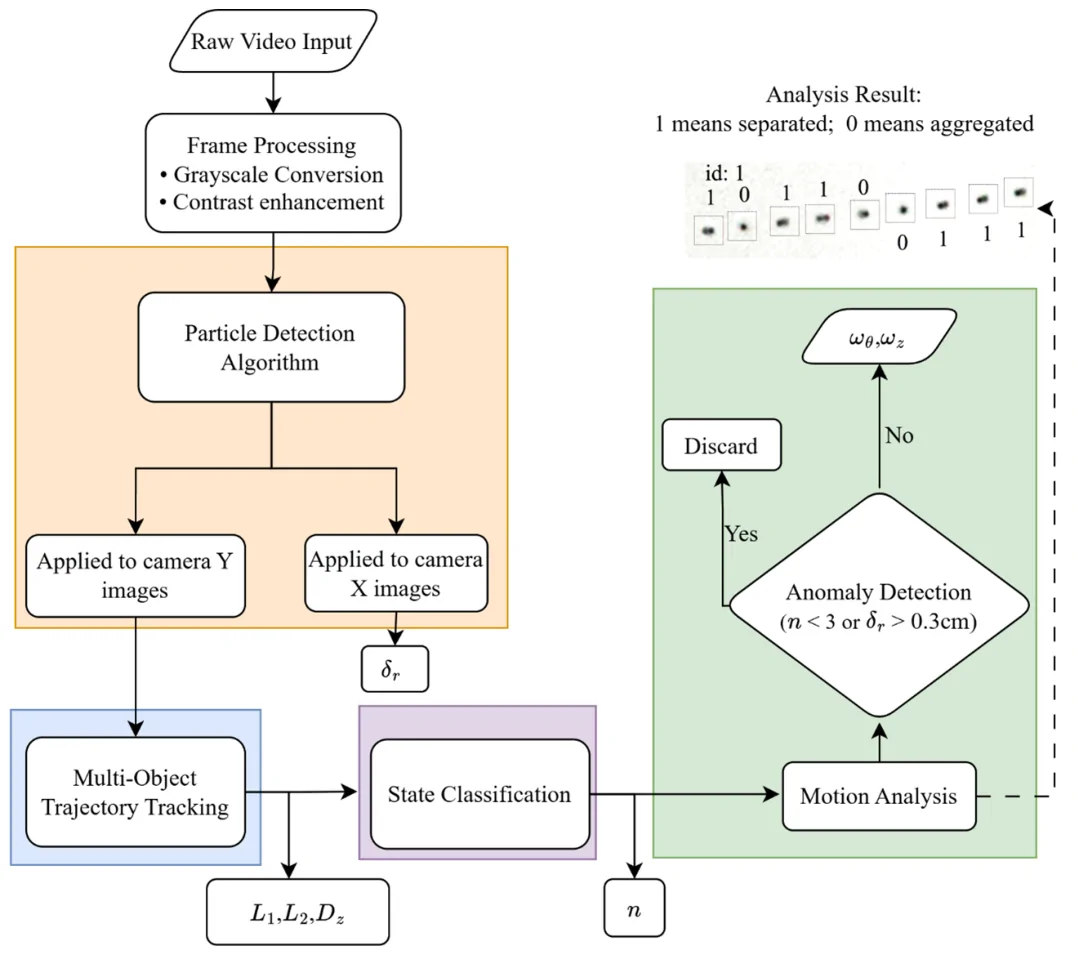
Flowchart of particle motion sensing pipeline. The colored panels denote the processing modules: orange for particle detection (applied to the Y- and X-camera images), blue for multi-object trajectory tracking, purple for motion-state classification, and green for motion analysis; the arrows indicate the data flow direction.

**Figure 4:**
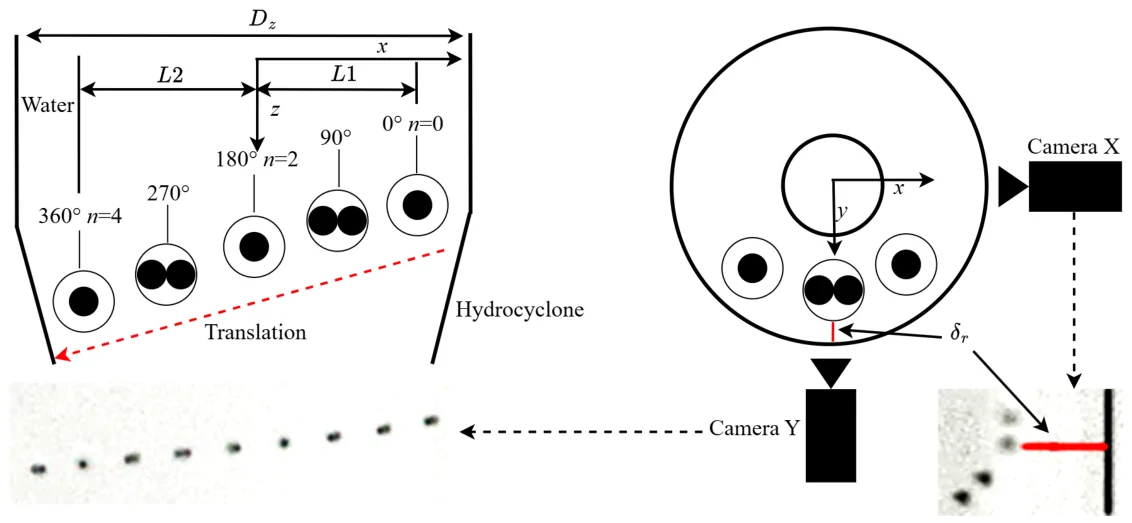
Physical Parameters Required for the Calculation of Particle Velocities.

**Figure 5:**
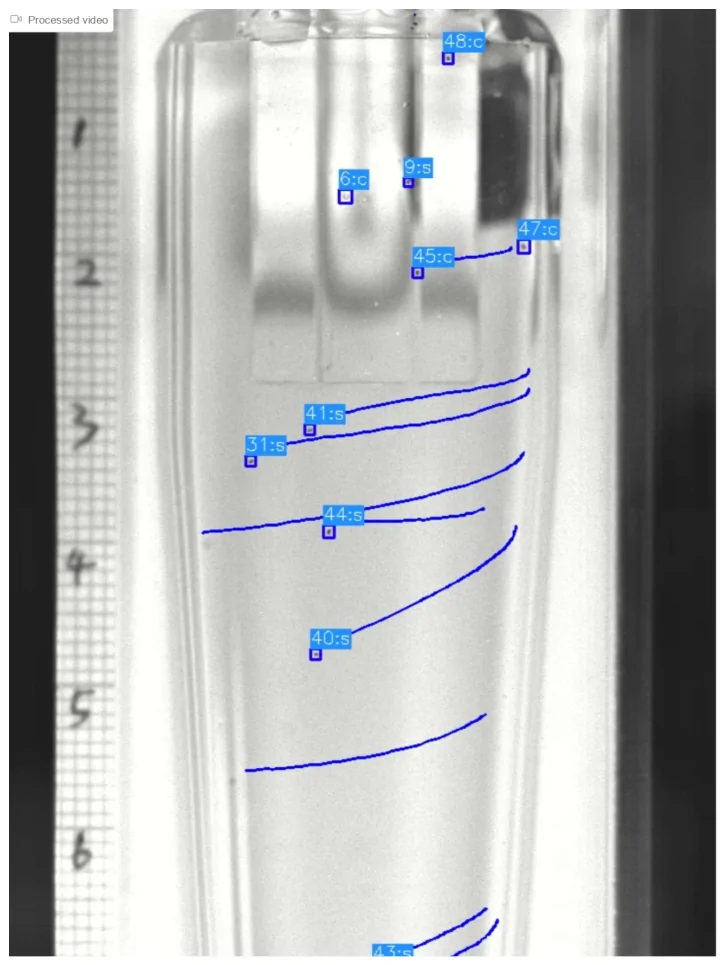
Visualization of particle tracking results on the processed high-speed video. Detected particles are enclosed by blue bounding boxes, and the blue curves represent the reconstructed trajectories of individual particles across consecutive frames. The tags beside each bounding box indicate the time stamp (in seconds) of the corresponding trajectory point in the processed video. The ruler at the left provides the spatial scale.

**Figure 6:**
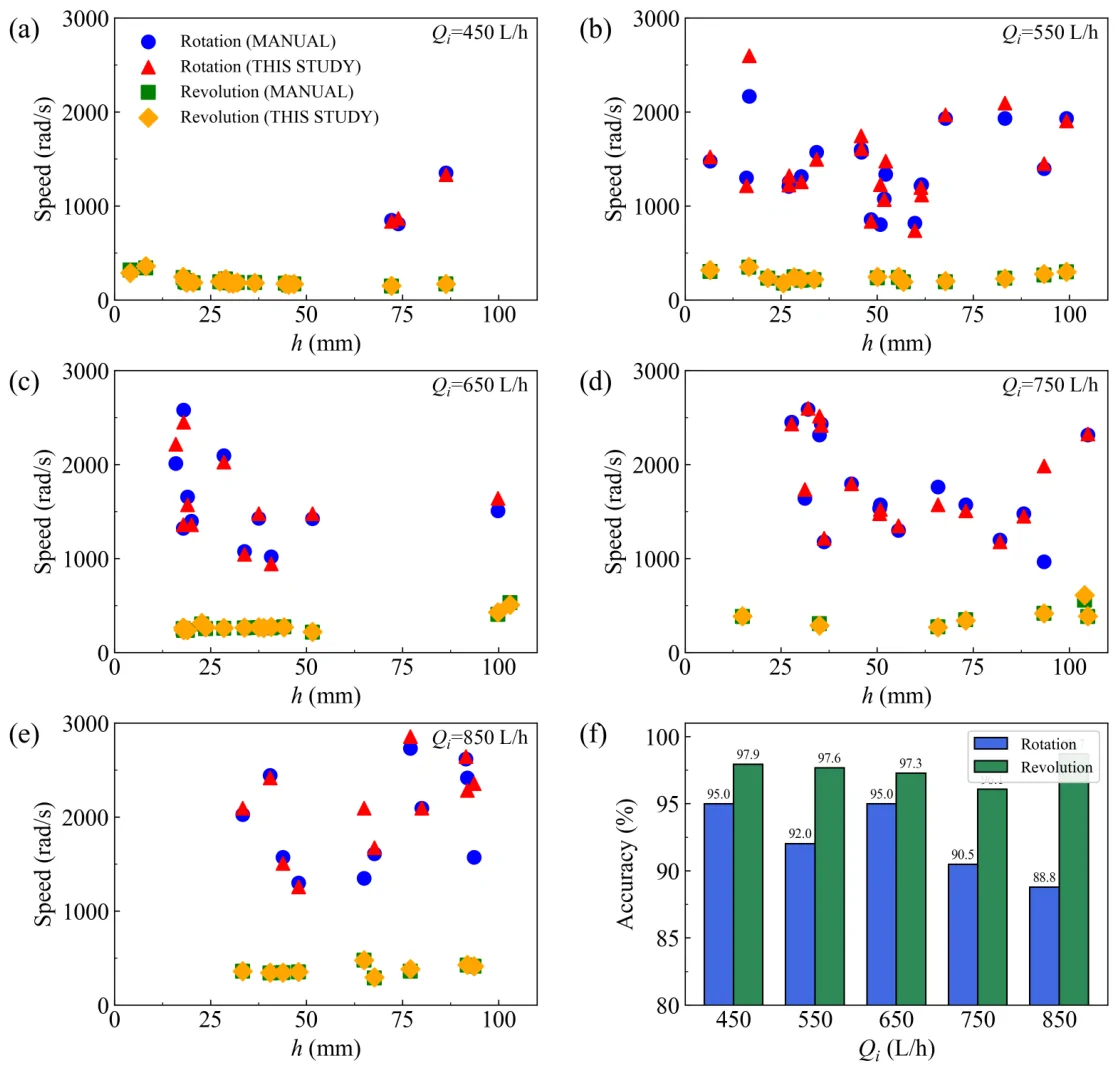
Accuracy validation of machine learning-based motion parameter measurement method. (**a**–**e**) Comparison of velocity distribution consistency between the proposed method (THIS STUDY) and manual measurement baseline (MANUAL) at different inlet flow rates; (**f**) accuracy of self-rotation and revolution velocity estimation at each inlet flow rate $Q_{i}$. Note: $Q_{i}$ denotes inlet flow rate; the horizontal axis *h* represents the axial height where the particle is located.

**Figure 7:**
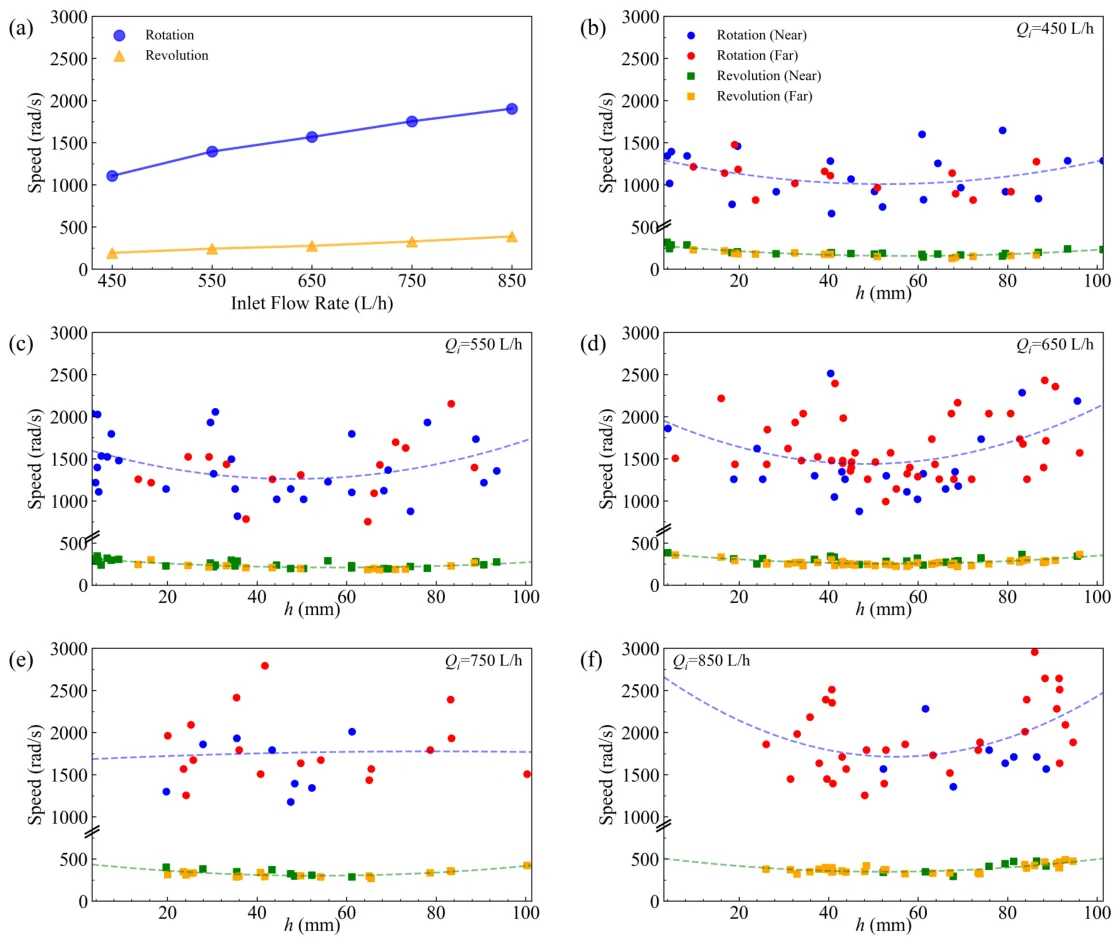
Particle self-rotation and revolution velocity distribution characteristics under different inlet flow rates. (**a**) Relationship of average velocity with inlet flow rate and (**b**–**f**) distribution of particle self-rotation and revolution speed variation with axial height; Note: $Q_{i}$ in the figure denotes inlet flow rate; in the legend, (Far) and (Near) represent the particle radial positions relative to the hydrocyclone wall. The classification is determined by the ratio of the particle revolution radius to the local diameter of the cylindrical or conical section at the corresponding axial position. A ratio > 0.9 is defined as the near-wall region (Near), while a ratio less than 0.9 corresponds to the central region (Far).

**Table 1:** Experimental setup information.

Component Name	Model	Manufacturer	Country of Origin
Camera 1	MotionXtra N4	IDT	Coralville, IA, USA
Camera 2	GigaView GVCC08-B05	SVSI	Huntsville, AL, USA
Macro lenses	AF Micro-NIKKOR 60 mm f/2.8D	Nikon	Tokyo, Japan

**Table 2:** Performance comparison of representative object detection frameworks.

Model	Precision	Recall	mAP@0.5	mAP@0.5:0.95	Inference Time
YOLOv5s	83.17%	82.92%	75.51%	30.57%	12.99 ms
YOLOv8s	84.62%	83.58%	79.84%	30.71%	14.36 ms
YOLOv9s	79.81%	81.28%	70.53%	27.98%	22.33 ms
RT-DETR-L	87.15%	90.15%	87.87%	41.15%	35.04 ms

**Table 3:** Tracking performance comparison between OC-SORT and ByteTrack.

Model	HOTA	MOTA
YOLOv8 + OC-SORT	50.2	64.986
YOLOv8 + ByteTrack	63.791	90.49

**Table 4:** Particle state classification performance.

Training Data	Test Accuracy	Performance Improvement
Real data only	98.62%	–
Real data + SAGAN augmentation	99.54%	+0.92%

**Table 5:** Comparison of particle detection and state-classification pipelines.

Method	Precision (%)	Recall (%)	Avg. Cost Time (ms)
YOLOv8 + OpenCV	73.77	72.16	16.97
Mask R-CNN	84.90	63.70	63.04
YOLOv8 + EfficientNet-B1	84.23	83.20	7.36

Note: the combined precision and recall of the YOLOv8-based pipelines are estimated from the corresponding stage-wise metrics as $P_{\mathrm{combined}}=P_{\det}\times P_{\mathrm{cls}}$ and $R_{\mathrm{combined}}=R_{\det}\times R_{\mathrm{cls}}$, where the detection stage uses the Y-view detector ($P_{\det}=84.62\%$, $R_{\det}=83.58\%$) and the classification stage is evaluated on the particle-state classification test set (222 aggregated-state and 214 separated-state images, macro-averaged over the two classes; OpenCV: 87.18%/86.34%, EfficientNet-B1: 99.54%/99.55%). Mask R-CNN is an independent pixel-level segmentation method without separately decomposable stages and therefore reports its own metrics on its own segmentation dataset.

**Table 6:** The calculation variables of particle velocities.

Variable	Symbol	Source/Calculation
Edge distance	δr	Edge distance in Camera X from YOLOv8 detections
Trajectory start frame	$F_{start}$	First frame index in the ByteTrack trajectory
Trajectory end frame	$F_{end}$	Last frame index in the ByteTrack trajectory
Number of frames in trajectory	$N_{f}$	$F_{end}-F_{start}+1$
Initial radial distance	$L_{1}$	Migration distance from centreline at first frame (Camera Y, via YOLOv8/ByteTrack)
Final radial distance	$L_{2}$	Migration distance from centreline at last frame (Camera Y, via YOLOv8/ByteTrack)
Axial position	$h_{start},\;h_{end}$	Axial heights at first and last frames (Camera Y, from tracking)
Representative diameter	$D_{z}$	Hydrocyclone diameter at $h_{avg}=\left(h_{start}+h_{end}\right)/2$
State-change count	*n*	Number of label transitions in the EfficientNet state sequence
Duration of each state	$T_{s}$	Number of consecutive frames for each state × frame interval

**Table 7:** Particle detection performance on test sets.

Model	Precision	Recall	mAP@0.5
Y-view Model	84.62%	83.58%	79.84%
X-view Model	69.23%	82.49%	71.62%

Note: the Y-view model was evaluated on the 65-image Y-camera test set; the X-view model, trained with the mixed-view strategy, was evaluated on the merged test set comprising 65 Y-camera and 36 X-camera images.

**Table 8:** Comparison of average processing time per particle trajectory.

Method	Average Time per Trajectory (s)
Manual annotation	600
Machine learning (this study)	11.1

## Data Availability

The raw data supporting the conclusions of this article will be made available by the authors on request. The training code, the data-split manifests, and a minimal sample dataset are openly available in GitHub at https://github.com/14790897/hydrocyclone-particle-measurement (accessed on 17 September 2026).
